# Full course macro-kinematic analysis of a 10 km classical cross-country skiing competition

**DOI:** 10.1371/journal.pone.0182262

**Published:** 2017-08-01

**Authors:** Finn Marsland, Colin Mackintosh, Hans-Christer Holmberg, Judith Anson, Gordon Waddington, Keith Lyons, Dale Chapman

**Affiliations:** 1 UC Research Institute for Sport and Exercise, University of Canberra, Bruce, Australian Capital Territory, Australia; 2 Australian Institute of Sport, Bruce, Australian Capital Territory, Australia; 3 APPSEN Pty Ltd, Canberra, Australia; 4 Swedish Wintersport Research Centre, Ostersund, Sweden; Norwegian University of Science and Technology, NORWAY

## Abstract

In this study micro-sensors were employed to analyse macro-kinematic parameters during a classical cross-country skiing competition (10 km, 2-lap). Data were collected from eight male participants during the Australian championship competition wearing a single micro-sensor unit (MinimaxX™, S4) positioned on their upper back. Algorithms and visual classification were used to identify skiing sub-techniques and calculate velocities, cycle lengths (CL) and cycle rates (CR) over the entire course. Double poling (DP) was the predominant cyclical sub-technique utilised (43 ± 5% of total distance), followed by diagonal stride (DS, 16 ± 4%) and kick double poling (KDP, 5 ± 4%), with the non-propulsive Tuck technique accounting for 24 ± 4% of the course. Large within-athlete variances in CL and CR occurred, particularly for DS (CV% = 25 ± 2% and CV% = 15 ± 2%, respectively). For all sub-techniques the mean CR on both laps and for the slower and faster skiers were similar, while there was a trend for the mean velocities in all sub-techniques by the faster athletes to be higher. Overall velocity and mean DP-CL were significantly higher on Lap 1, with no significant change in KDP-CL or DS-CL between laps. Distinct individual velocity thresholds for transitions between sub-techniques were observed. Clearly, valuable insights into cross-country skiing performance can be gained through continuous macro-kinematic monitoring during competition.

## Introduction

Cross-country skiing is unique in that athletes alter between distinct sub-techniques frequently within a single event in order to optimise speed and efficiency over varying terrain. Swimming is the only other major sport involving distinct sub-techniques, combined only in the individual medley with transitions at set distances. Although in longer freestyle technique events swimmers may strategically change between 2, 4 or 6 kicks per stroke [1], in shorter events they commonly retain the same kick rate relative to stroke. Cross-country skiers have been observed to change sub-techniques more than 30 times in a 1.4 kilometre sprint event [2].

The three major cyclical sub-techniques of classical cross-country skiing are double poling (DP), kick double poling (KDP) and diagonal stride (DS), with other alternate arm/leg techniques such as herringbone or “diagonal running” without gliding [3]. Furthermore, with sufficient velocity on downhills the tucking (Tuck) technique is used [2], and when turning corners a wide variety of sliding, stepping and poling techniques (Turn) may be utilised, dependent on velocity, turn radius and personal preference [4]. While minor adjustments in joint angles or timing of force application can improve efficiency [5, 6], the macro-kinematic parameters cycle rate (CR), cycle length (CL) and choice of sub-technique are the main determinants of performance.

International race course guidelines [7] are general enough to allow every event to include its own distinct combination of uphills, downhills and flat/undulating terrain of varying gradients. Together with complex variations in ski-snow friction due to air and snow temperature, humidity, crystal size and age [8], these course variations reduce the usefulness of comparing performance times in different races. Consequently, analysis of cross-country skiing performance in competitions has traditionally been limited to comparisons between overall times, lap times or split times for sections of the course within a single race. Although often measured in the laboratory [9–12], CR and CL are seldom determined during daily training or competition, and, even then, typically only for sections of a course to obtain snapshots of kinematics [13–16].

Of the numerous studies in both the laboratory and field that have examined CR and CL for the different classical techniques, most have involved pre-determined sub-techniques and/or stepwise increases in treadmill gradient and/or speed. On a treadmill it is difficult to simulate the variation in terrain and changes in direction that occur during competition on snow, and field analyses such as performed by Andersson and colleagues [2] have provided useful insights.

Recent studies have demonstrated that micro-sensor technology can be used to monitor performance throughout an entire race or training session in the field. Myklebust et al. [17] and Marsland et al. [18] analysed such data collected on snow, while Sakurai and associates [19, 20] have developed algorithms that allow analysis of macro-kinematics collected from rollerskiing outdoors, laying the groundwork for monitoring entire competitions. As Bolger and colleagues [21] noted, analysis of macro-kinematic variables would greatly improve our knowledge of what contributes to performance.

The present study used micro-sensors to identify sub-techniques of cross-country skiing and to measure macro-kinematics over the entire length of a distance competition. We hypothesised that there would be differences in technique use and cycle characteristics throughout the race, between laps, and between faster and slower competitors. Velocity thresholds for transitions between the different techniques were also anticipated, despite individual preferences.

## Materials and methods

### Participants

The physical characteristics and FIS points of the eight male participants in the study are shown in Table 1. Data were collected during an Australian Cross-Country Skiing Championship event at Falls Creek. Ethical approval for the study was obtained from the University of Canberra Committee for Ethics in Human Research (approval number 13–113). All participants were well informed about the study and given the opportunity to ask questions prior to providing signed consent.

**Table 1:** Characteristics of the participants (mean ± s, n = 8).

Age (years)	27.0 ± 7.1
Body height (cm)	182.0 ± 5.6
Body weight (kg)	77.1 ± 7.0
FIS points, distance	129.4 ± 64.7
FIS points, sprint	140.0 ± 81.4
VO2 max (ml ∙ kg^-1^ ∙ min^-1^)	73.4 ± 6.7

### Equipment

Kinematic data were collected using micro-sensor units (67 g; 2.0 × 4.8 × 8.5 cm; MinimaxX^™^ S4, Catapult Innovations, Melbourne, Australia) positioned centrally on the upper-back using a lightweight cloth harness underneath a standard competition number. These units contained a triaxial accelerometer (100 Hz, ± 6 g), a gyroscope (100 Hz, ± 17.5 rad·s^-1^), and a Global Positioning System (GPS) device (Fastrax, 10 Hz). The accelerometer was configured vertically [22], and the accelerometer and gyroscope components calibrated prior to data collection. Using a cradle supplied by the manufacturer and connected to a personal computer each micro-sensor unit was held in position while the direction of the three acceleration axis were set, following which the unit was rotated 90° around each axis to quantify angular acceleration [23].

### Study design

Data were collected on a FIS homologated track (registration number 09/22.03/05, total climb 156 m, maximum climb 32 m, height difference 53 m). The nominal 10 km event took place on an approximately 5.5 km loop, with some minor adjustments from the homologated course due to snow conditions. The participants warmed up employing their own personal routines and were seeded according to their current FIS and Australian rankings. The competition was held in accordance with FIS rules, using a 30 second start interval. The air and snow temperature were recorded at the start and finish of the event. In general the snow was well packed and firm, and all participants experienced similar conditions. There was no standardisation of ski equipment or ski wax, allowing participants to use and wax their own skis together with their personal supporters.

### Technique classification

Micro-sensor data were downloaded to a laptop computer and imported into analysis software (Makesens V73.0, Appsen, Canberra, Australia). An algorithm involving a low-pass Butterworth filter (gyroscope and accelerometer signals were filtered with a cut-off frequency of 1.0 Hz and 2.0 Hz, respectively) was applied to classify the technique cycles and sections for each of the sub-techniques automatically. The algorithm as described by Marsland et al. [18] was used to classify cyclical sub-techniques, with minor modifications in Turn and Tuck detection to improve overall detection rates. The same algorithm was applied to the micro-sensor for all athletes. This processed data was then examined visually for errors in classification, using a graphical representation of all six filtered accelerometer and gyroscope signals to compare movements from each athlete with typical sub-technique patterns, and to confirm the magnitudes of acceleration in each identified cycle matched the classified sub-technique.

The universal cyclical classical sub-techniques classified were double poling (DP), kick double poling (KDP), and diagonal stride (DS), and non-cyclical techniques tucking (Tuck) and turning (Turn). Herringbone and any similar “diagonal running” technique without glide were classified as DS, due to the challenge of differentiating between these sub-techniques. A DP cycle contained one double poling action, a KDP cycle contained one double poling action and one kick action (from either leg) and a DS cycle contained a poling action and a kick action from each arm and leg in diagonal style (starting from either side). If there was uncertainty as to the sub-technique used, or partial cycles or irregular technique such as transitions between sub-techniques observed, the technique was classified as Misc. Technique was deemed to have been correctly classified by the algorithm if no change was made to the algorithm classification after visual examination, the percentage accuracy was calculated by dividing the number of correct algorithm classifications by the total number of classifications. The visual classifications were made by a cross-country skiing coach with four years of experience examining corresponding micro-sensor and video data. The intra-rater reliability for the combined algorithm and visual classification check was very high for mean DP, KDP and DS cycle velocity, cycle length and cycle rate (ICC = 1.0 (CL = 0.99–1.00), CV% = 0.07–0.55) and high for total DP, KDP and DS cycle count, distance and time (ICC = 0.99–1.00, CV% = 0.44–2.65).

Using the micro-sensor unit GPS data, velocity was calculated using the Doppler shift method, with distance calculated by differentiating the velocity over time as described by Marsland et al. [18]. Mean velocities for each sub-technique are calculated from all individual sub-technique cycles (and instances of Tuck, Turn and Misc). Athletes’ overall performance was ranked based on velocity. Velocity was calculated by dividing the mean GPS lap/race distance for all athletes by the lap/race time for each athlete. The best estimate of the true course distance was the mean GPS distance, as changes were made to the homologated course due to snow conditions.

### Statistics

All macro kinematic data for each sub-technique and lap were determined to be normally distributed using the Shapiro-Wilk test. Paired t-tests were used to compare Lap 1 and Lap 2, with mean differences (MDiff%) and 95% confidence intervals (CI) presented as percentages. Coefficients of variation (CV%) for within-athlete variations in cycle parameters (CL, CR and velocity) were calculated and operationally defined as small (< 5%), moderate (5–10%) or large (> 10%) variations based on prior experience. Data from the four fastest and four slowest participants based on overall race time were visually compared for trends in mean cycle parameters. Statistical analyses were performed using Prism (GraphPad) and Excel (Microsoft), with an alpha level of *P* <0.05, and means are presented as mean ± standard deviation (*s)*. Cohen’s *d* effect size (ES) for comparisons between laps or groups were calculated and classified as trivial (0.0–0.2), small (0.2–0.6), moderate (0.6–1.2), large (1.2–2.0), and very large (> 2.0).

## Results

### Overall performance

**Fig 1:**
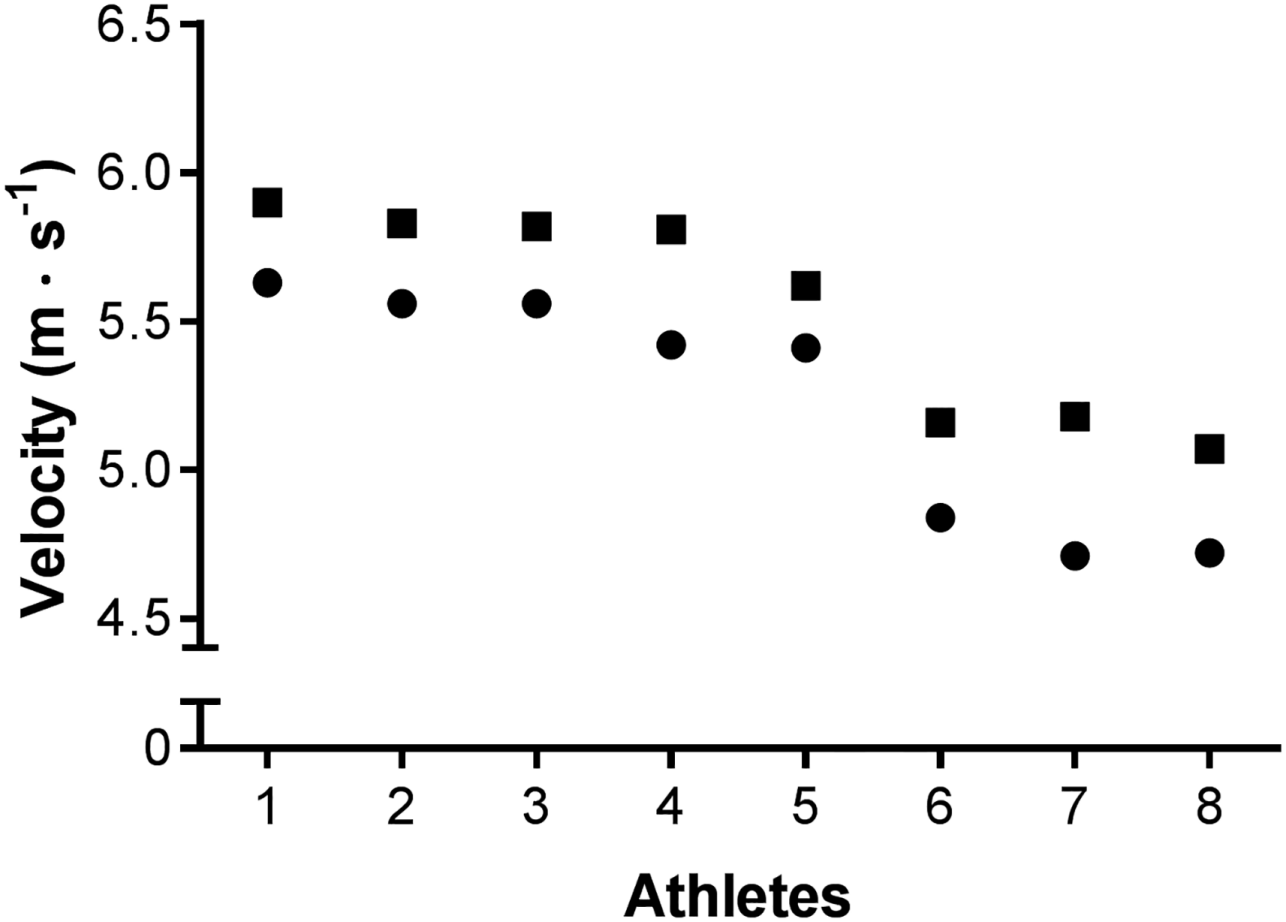
The mean race velocities with which each athlete completed Lap 1 (■) and Lap 2 (●). 1–8 = fastest—slowest (entire race).

The mean velocity to complete the full competition was 5.38 ± 0.36 m∙s^-1^ (5.54 ± 0.34 m∙s^-1^ first lap, 5.24 ± 0.39 m∙s^-1^ second lap), with mean velocities for individual laps ranging from 4.74 to 5.86 m∙s^-1^ (Fig 1). On average, each athlete covered 11055 ± 101 m (Lap 1, 5517 ± 54 m; Lap 2, 5538 ± 49 m), and the mean completion time was 34:22 ± 2:29 min (range 31:59–37:41). The first lap was completed significantly faster than the second (16:40 ± 1:05 versus 17:42 ± 1:24 min, MDiff% = 1.50, 95% CI = 1.10–1.88, ES = 0.06, P < 0.0001).

### Technique usage

All skiing was classified into cyclical and non-cyclical sub-techniques, with a small proportion (6.7 ± 2.1%, by distance) of irregular motion classified as Misc (Table 2). Prior to manual classification 90.2 ± 2.9% (range 84.1–94.2) instances of sub-technique classifications (cycles or sections of Turn or Tuck) were determined accurately classified by the algorithm. Athletes changed sub-technique 279 ± 18 times (25.4 ± 1.7 times per km), corresponding to technique change every 40 m, with less than 10 s between changes. In terms of both distance and time, DP was utilised the most, followed by Tuck, DS, KDP and Turn, respectively. There was a trend for athletes to use DS to a greater, and KDP to a lesser, extent on Lap 2 relative to Lap 1. In terms of both distance and time, the amount of DP, Tuck and Turn used were similar on both laps.

**Table 2:** Sub-technique usage (mean ± *s*) by the 8 athletes.

Sub-technique	% of the distance (m) (% of the time (s))		
	Entire race	Lap 1	Lap 2
DP	42.8 ± 5.2 (40.5 ± 6.2)	43.2 ± 4.4 (41.1 ± 5.5)	42.4 ± 6.2 (39.9 ± 7.1)
KDP	5.5 ± 4.1 (6.7 ± 5.1)	6.3 ± 4.7 (7.9 ± 5.9)	4.6 ± 4.0 (5.6 ± 5.1)
DS	16.1 ± 4.0 (24.9 ± 5.6)	14.7 ± 3.4 (23.2 ± 4.9)	17.4 ± 5.2 (26.5 ± 6.8)
Tuck	24.3 ± 4.1 (15.9 ± 3.0)	24.8 ± 4.5 (16.2 ± 3.2)	23.9 ± 3.9 (15.7 ± 3.0)
Turn	4.6 ± 0.6 (4.5 ± 0.7)	4.7 ± 0.6 (4.7 ± 0.6)	4.5 ± 0.8 (4.3 ± 0.8)
Misc	6.7 ± 2.0 (7.4 ± 2.0)	6.2 ± 1.9 (6.9 ± 1.8)	7.2 ± 2.2 (7.9 ± 2.2)

DP = double poling; KDP = kick double poling; DS = diagonal stride; Tuck = tucking; Turn = turning; Misc = all other techniques.

### Kinematics

The fastest sub-technique was Tuck, followed by DP, Turn, KDP and DS (Table 3). There was a small but significant drop in velocity from Lap 1 to Lap 2 for DP (MDiff% = 1.06, 95% CI = 0.63–1.49, ES = 0.04, P < 0.001) and Tuck (MDiff% = 1.44, 95% CI = 1.05–1.84, ES = 0.06, P < 0.01), with no differences observed for other sub-techniques. There was a small decrease in CL for DP on Lap 2 relative to Lap 1 (MDiff% = 1.15, 95% CI = 0.58–1.73, ES = 0.05, P < 0.01), with no significant difference in CL for DS or KDP. The CR for Lap 1 and Lap 2 were similar for all sub-techniques.

**Table 3:** The velocities, cycle lengths and cycle rates (mean ± *s*) for the various sub-techniques.

Technique	Velocity (m∙s^-1^)			Cycle length (m)			Cycle rate (cycle∙min^-1^)		
	Entire race	Lap 1	Lap 2	Entire race	Lap 1	Lap 2	Entire race	Lap 1	Lap 2
DP	5.7 ± 0.5	5.8 ± 0.5	5.6 ± 0.5 *	6.3 ± 0.8	6.5 ± 0.8	6.2 ± 0.8 *	55.1 ± 6.0	55.1 ± 6.4	55.2 ± 6.0
DS	3.4 ± 0.3	3.5 ± 0.3	3.4 ± 0.3	3.6 ± 0.4	3.7 ± 0.4	3.6 ± 0.4	58.8 ± 3.4	58.9 ± 3.2	58.7 ± 3.7
KDP	4.4 ± 0.4	4.4 ± 0.5	4.4 ± 0.2	5.7 ± 0.5	5.7 ± 0.6	5.8 ± 0.3	45.8 ± 2.5	46.1 ± 2.4	45.5 ± 2.7
Tuck **	8.2 ± 0.4	8.5 ± 0.3	8.0 ± 0.4 *	90.1 ± 15.9	93.3 ± 18.0	86.9 ± 14.0	‒	‒	‒
Turn	5.5 ± 0.4	5.6 ± 0.4	5.4 ± 0.3	‒	‒	‒	‒	‒	‒
Misc	4.8 ± 0.4	4.9 ± 0.4	4.7 ± 0.3	‒	‒	‒	‒	‒	‒
Overall	5.4 ± 0.4	5.5 ± 0.3	5.2 ± 0.4 *	‒	‒	‒	‒	‒	‒

DP = double poling; KDP = kick double poling; DS = diagonal stride; Tuck = tucking; Turn = turning; Misc = all other techniques; ‒ = not relevant.

* *P* < 0.01 for Lap 1 in comparison to Lap 2

** The cycle length values for Tuck are the mean distance travelled for each usage of this non-cyclical technique for each participant.

### Within-athlete cycle variability

**Fig 2:**
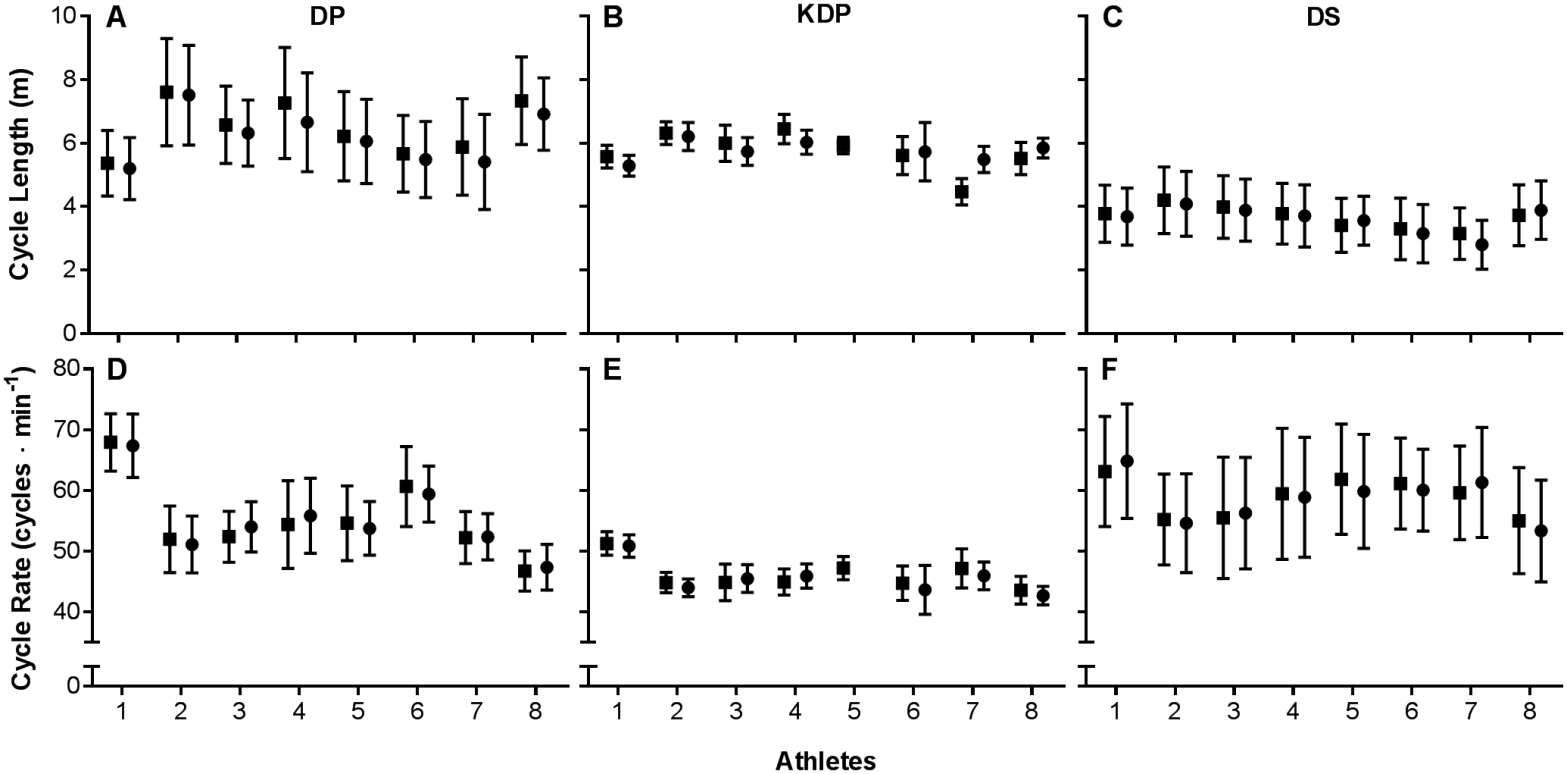
The mean cycle lengths and rates (± *s*) for each athlete and cyclical sub-technique on Lap 1 (■) and Lap 2 (●). 1–8 = fastest—slowest (entire race); DP = double poling; KDP = kick double poling; DS = diagonal stride.

**Fig 3:**
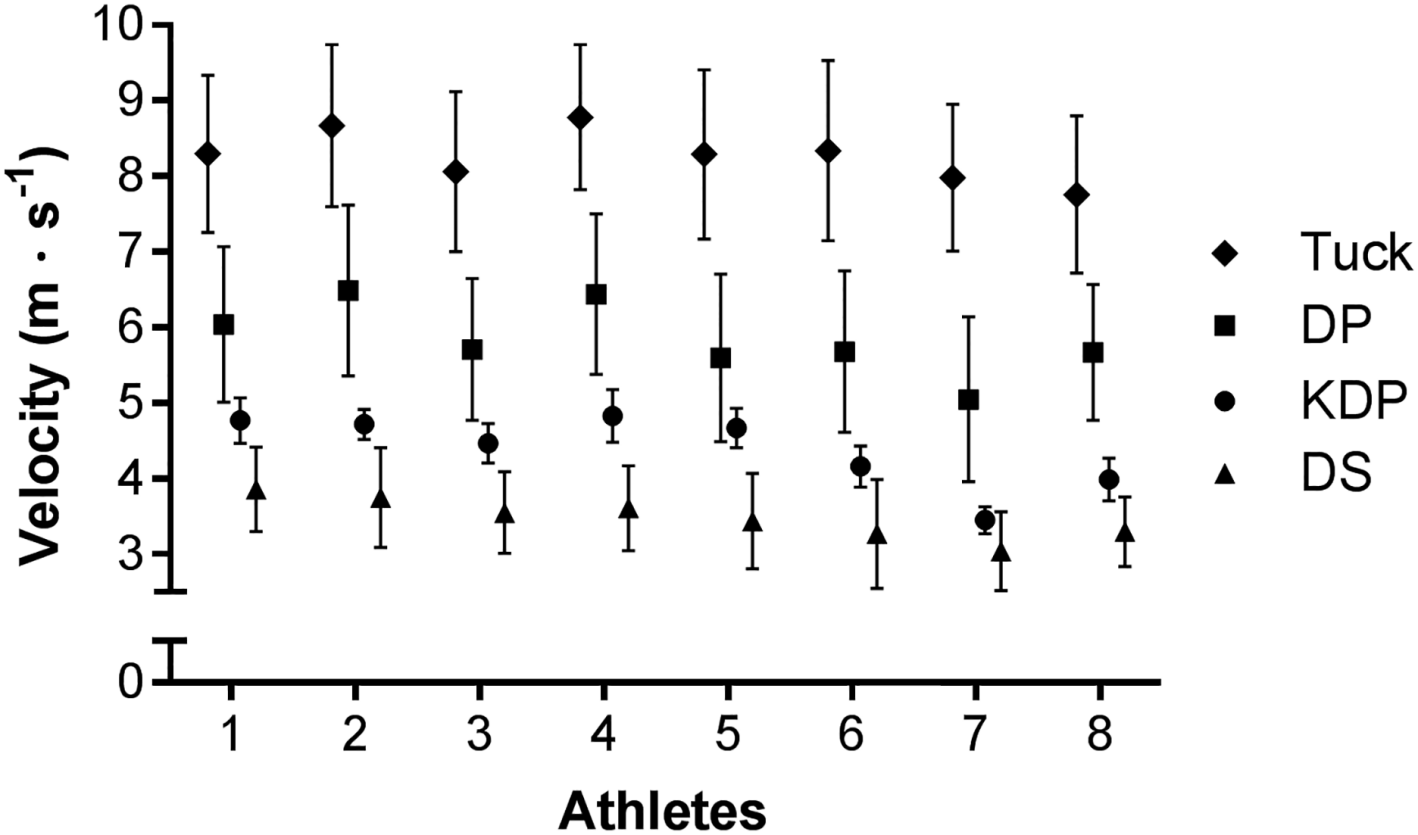
The mean velocities (± *s*) for each athlete for the various sub-techniques on Lap 1. 1–8 = fastest—slowest (entire race); Tuck = tucking; DP = double poling; KDP = kick double poling; DS = diagonal stride.

The mean cycle lengths and cycle rates for each participant, sub-technique and lap are shown in Fig 2. Within-athlete variability in CL was large for DS (CV% = 25 ± 2%) and DP (CV% = 21 ± 3%), and moderate for KDP (CV% = 8 ± 3%). With respect to CR, this variability was large for DS (15 ± 2%), moderate for DP (CV% = 9 ± 2%) and low for KDP (CV% = 5 ± 2%). Within-athlete variability in velocity (± *s*) was large for DP (CV% = 18 ± 3%) and DS (CV% = 17 ± 3%), and moderate for KDP (CV% = 6 ± 1%) (Fig 3). The CV% values are for all laps, n = 16 (n = 15 for KDP).

### Faster versus slower athletes

**Fig 4:**
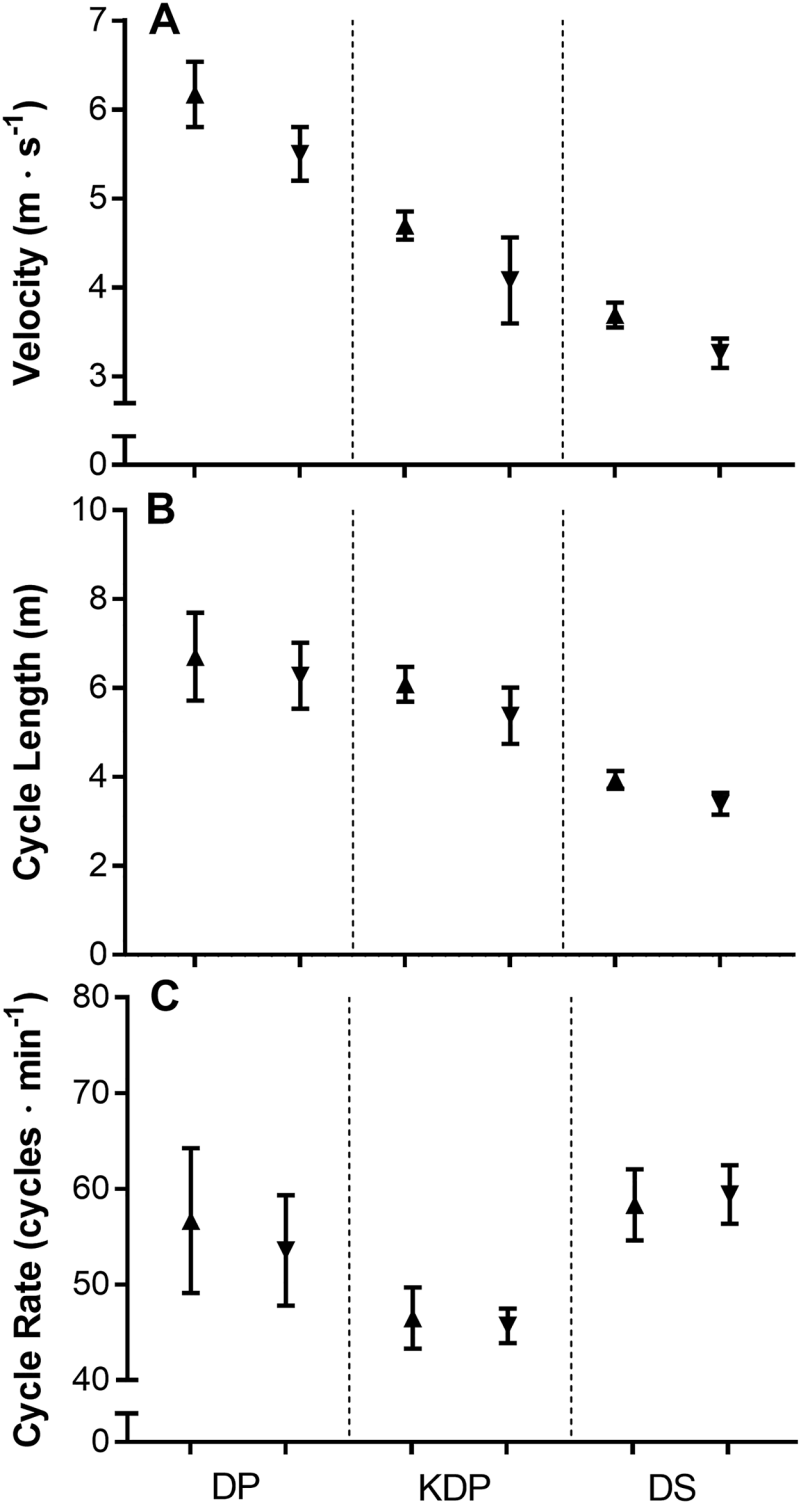
The mean velocities, cycle lengths and cycle rates (± *s*) for the fastest four (▲) and slowest four (▼) athletes in each cyclical sub-technique. DP = double poling; KDP = kick double poling; DS = diagonal stride.

Mean overall velocity of the fastest four athletes was 5.8 ± 0.1 m∙s^-1^, versus 5.2 ± 0.1 m∙s^-1^ for the slowest four. There was a trend for the mean velocities for the faster athletes to be higher with all sub-techniques, while mean CL was longer only for DS compared to slower athletes, and mean CR for all athletes and sub-techniques were similar (Fig 4).

## Discussion

The major findings of this first study to undertake a macro-kinematic analysis of an entire classical cross-country skiing competition on-snow using micro-sensors were as follows. 1) The locomotion over 93.3 ± 2.0% of the race distance could be identified as one of the standard classical sub-techniques. The cyclical sub-technique employed most extensively was DP, with a moderate amount of DS and minimal KDP. The non-propulsive Tuck technique was utilised more than expected, accounting for almost 1/4 of the distance covered and 1/6 of the race time. 2) A wide range of velocities, CLs and CRs were utilised by individuals. 3) The CL was significantly longer on Lap 1 with the DP sub-technique, and overall mean velocity higher, with no significant difference in CL between laps with the other cyclical sub-techniques. The CR was similar on both laps for all sub-techniques. 4) The choice of technique appeared to be related to velocity, with transition thresholds observed for each individual. 5) There was a trend for faster athletes to be faster for all sub-techniques, noting that this was achieved with different combinations of CL and CR. Similar CR were observed for the faster and slower athletes.

### Technique usage

Classifying sub-techniques and measuring kinematic parameters throughout an entire race is an important progressive step for cross-country skiing performance analysis. Snapshot analyses using video technology are incomplete and to date little is known about the relative proportions of sub-techniques used during competition. The difficulties in comparing to other published literature involving technique classification include the lack of literature on classical technique detection over a full course on-snow and no other sports have the range of sub-techniques and frequency of transitions that cross-country skiing has. Of interest, Sakurai et al. [20] reported a 98.5% detection accuracy on rollerskis in a classical technique time-trial using a four micro-sensor arrangement, however, unlike on snow, rollerskiing tends not to be performed on courses with technical (sharp) corners. While on snow, Stoeggl et al. [24] reported an 86.0% accuracy of detecting skating technique using a single micro-sensor (accelerometers in a mobile phone), which was improved to 90.3% with machine learning from individual data.

The high proportion of DP use observed in our study was not unexpected, since in recent years improvements in upper body power and endurance [25] allow athletes to utilise DP up increasingly steeper inclines. For the first time in 2015 the winner of a 10 km classical World Cup race used only DP and tucking and turning techniques [26]. As Göpfert and co-workers [5] noted, in an attempt to limit the use of DP in sprint races FIS modified their homologation guidelines to increase the amount of vertical elevation. FIS rule adaptations have gone further in 2016 [27, 28], with race organisers now permitted to ban DP in certain sections of the track, and the introduction of pole length restrictions to limit advantages from using only DP [29].

The relative low proportion of KDP recorded in this study is consistent with observations reported by Göpfert et al. [5]. Furthermore, we observed large variations in individual use of KDP, with athlete 5 using no KDP on Lap 2 (Fig 2B), athletes 4 and 8 using nearly half as many cycles on Lap 2, and athlete 6 using only two KDP cycles on Lap 2. The slowest sub-technique was DS, used for 16% of the distance but 24% of the time, primarily on steeper sections. Although this proportion appears to be in contrast to the 56% of time spent on uphills reported by Bolger and co-workers [21], their values are a composite of DS, KDP, and DP. Our methodology enables uphill time to be classified into all three cyclical sub-technique components.

The relatively high use of Tuck (24% of total race time) may have implications for training. The longest continuous usage of this technique was 18–43 s, with one competitor tucking for 1753 out of 5480 m on the first lap. Bolger et al. [21] reported that 27% of time spent on downhill sections during a 15 km classical competition, but the sub-techniques used were not identified and might have involved propulsive techniques. We also found that Tuck was used on flat sections after downhills until the skiing velocity dropped sufficiently to induce a transition to DP. Typically during interval training coaches simulate race scenarios with low intensity exercise between repetitions [30], however to retain greater ecological validity during training the instruction should be to hold the Tuck position for durations relevant to competitions during recovery periods.

### Cycle kinematics

The high within-athlete variability of CL and CR shown in Fig 2A–2F likely reflects the varying terrain, with higher cycle rates on steeper sections of the track as reported by Sandbakk et al. [31] when studying freestyle techniques. This observation is also supported by visual examination of the cycle frequency on moderate and steeper uphill sections. While it is possible that different pacing strategies or effects of fatigue could have exerted an impact in this context, the within-athlete variability was almost the same for Lap 1 and Lap 2. Though the distance was shorter, Vesterinen and co-workers [32] also found no differences in cycle variables between heats in a simulated sprint competition on rollerskis.

Overall, the range of CR and CL for all of our athletes varied considerably, from 45–50 cycles·min^-1^ for DS-CR and up to 7–8 m for DP-CL. Although no other studies have monitored CR and CL continuously throughout an entire distance race on-snow, Sakurai and colleagues [20] observed large differences in sub-technique velocity during a distance competition on classic rollerskis. This wide range of CR utilised during competition reinforces variable frequency-based training methods that are already in use [12, 33, 34], while the ranges of CR and CL underline the benefits of training in varied terrain.

Earlier reported values of CL and CR from competition have typically involved a small data collection window. Nonetheless, our reported mean DS cycle kinematics are similar to the 57–66 cycles·min^-1^ and 3.5–4.4 m at 3.2–4.0 m∙s^-1^, reported by Bilodeau et al. [15] in competition, and the 52–59 cycles·min^-1^ and 4.0–4.5 m at 3.5–4.5 m∙s^-1^ as measured by Andersson and colleagues [35] at medium to high intensity. In contrast, the DP cycle kinematics from Bilodeau et al. [15] of 53–63 cycles·min^-1^ and 7.5–8.6 m at 6.8–8.0 m∙s^-1^ indicate similar CR but much higher CL and velocities than those observed with micro-sensors in the present study. While many uncontrolled variables influence gliding friction and ski speed, the key difference between this latter study and ours is that the DP kinematics were derived from only one 30 m section of flat terrain, compared to 4733 ± 585 m of DP collected over varied terrain in our case.

With a mean of 45.8 cycles∙min^-1^ at 4.4 m∙s^-1^ the KDP cycle rates were substantially lower than for DS and DP. As pointed out by Smith [36], this is likely due to the two-segment nature of the KDP movement pattern. CR and velocity in this study are in the same ranges as those observed by Smith [36] on-snow (48.0 cycles∙min^-1^ at 5.4 m∙s^-1^). Although Göpfert et al. [5] reported a KDP-CR of 23.3 cycles ∙ min^-1^ at 5.3 m∙s^-1^ on rollerskis, their definition of a cycle involved two poling cycles with a kick from each leg, so the rate for a single cycle is in the same range as reported here.

For all participants, the CL with DP was slightly, but significantly shorter, with no change between laps in the CL for DS or KDP. In this context the proportional use of each sub-technique should be taken into consideration: the mean use of KDP fell from 6.4% to 4.7% from Lap 1 to Lap 2, while that of DS rose from 14.7% to 17.4%. This switch in sub-technique also helps to account for the drop in overall race velocity, despite the lack of any change in the mean KDP and DS velocities. Andersson et al. [2] reported fewer transitions on the second lap in a simulated on snow sprint competition, but during our distance competition we observed no difference in the number of transitions per lap.

Although macro-kinematic measurements will vary according to terrain, the ability to measure sub-technique velocity, CL and CR on a particular race course under known snow conditions is potentially very useful for course profiling. It is common for athletes to prepare for World Championship and Winter Olympic competition by training on simulated courses, mimicking the distribution, length and gradient of uphills [37, 38]. The macro-kinematic and sub-technique distribution information provided here should improve such simulation.

### Velocity thresholds

Velocity thresholds for transitions between sub-techniques were seen in this study (Fig 3), in similar manner to the observation of thresholds in a simulated sprint in freestyle technique [2]. Sakurai and colleagues [20] also observed that during a 6.9 km time trial athletes on rollerskis selected classical sub-techniques on the basis of skiing velocity and course grade, with speeds ranging from 3.9–5.4 m·s^-1^ for DS, 2.0–7.3 m·s^-1^ for KDP, and 4.5–10.2 m·s^-1^ for DP. These latter two ranges recorded over an entire rollerski race also reflect the kinematic variability observed in the current study. On snow, Bolger and colleagues [21] described mean velocities of 4.8 m·s^-1^ on uphill sections and 7.2 m·s^-1^ on flat terrain, but the sub-techniques used and range of velocities on each section were not reported.

Hypothesising that transition thresholds in classical skiing are connected to poling forces, Pellegrini and colleagues [39] examined eight different inclines and six different speeds of rollerskiing on a treadmill and concluded that a variety of triggers are involved. With current technology poling forces cannot be measured during competition. It seems likely that skiers take both perceived velocity and perception of effort to maintain that velocity into consideration when deciding to change sub-technique. Velocity thresholds may help to determine the most efficient technique for a given speed and terrain, however this needs to be explored further. When skiers opt to compete in classical events without grip wax, they use DP over the velocity thresholds for KDP and DS on all but the steepest uphills (where Herringbone is employed), utilising shorter cycle lengths and higher cycle rates to maintain velocity as described by Sandbakk, Ettema and Holmberg [40] in freestyle technique. While DP may not be the most efficient technique on sections where skiing speed is above the normal DP velocity thresholds, this is compensated for by having faster skis on downhill and flat sections, as noted by Stöggl and Holmberg [25].

### Comparison between faster and slower skiers

In a simulated classical sprint competition, Stöggl and co-workers [38] observed that faster skiers exhibited longer CL for the same CR. In contrast, we observed high individuality in athletes’ strategy to achieve higher sub-technique velocities. For example, the fastest athlete used a higher CR and shorter CL for all three cyclical sub-techniques than most other athletes, while the second fastest athlete had one of the lowest mean CR and longest CL (Fig 2). While there was a trend for faster skiers to exhibit higher mean CL for DS, the proportion of sub-technique use also had an impact on this outcome and further research is required.

Interestingly, the two participants with the highest CR (athletes 1 and 6), also had the lowest body mass. Indeed, Stöggl et al. [41] found a relationship between upper-body muscle mass and peak speed in classical rollerskiing, while Hegge et al. [42] concluded that greater muscle mass contributes to kinematic differences between genders. This high CR by lighter skiers could be a conscious or sub-conscious strategy to compensate for shorter CL. Stöggl and colleagues [33] demonstrated using rollerskis on a treadmill that skiers who can apply greater force through their poles are able to ski at lower CR. Where natural CR for a given athlete are already high, future gains in skiing speed using a particular sub-technique may require increased emphasis on increasing CL as the ability to further increase CR may be limited.

### Future directions

Measuring cross-country skiing macro-kinematics over entire competitions and comparing between athletes, events, locations, and across different snow conditions, will greatly assist evaluation of individuals’ strengths and weaknesses and enable world’s best practice comparisons. Adaption of macro-kinematic analyses from classical to freestyle cross-country skiing sub-techniques is a logical development. With future technology, real-time macro-kinematic data would provide another dimension for spectators of international cross-country ski racing, in similar fashion to the way that heart-rate monitoring is currently used with live TV performance tracking. Recreational cross-country skiers and amateur racers may also find value in comparing macro-kinematic values from their own activities with those of acquaintances or against world elite.

## Conclusions

Macro-kinematic data collected continuously throughout a competition by a single micro-sensor unit provides new insight into cross-country skiing performance. The range and variability of velocities, cycle lengths, cycle rates indicate that the mean cycle kinematics must be considered in relationship to sub-technique distribution. While some key findings support and extend published observations, the extent of Tuck usage and variability in cycle kinematics are novel. Practical implications include the importance of training in varied terrain and utilising a wide range of CR both on and off-snow, greater focus on the use of Tuck in training to match the demands of competition, and tailoring training for individuals based on strengths and weaknesses highlighted by their sub-technique use. Further evaluation of sub-technique usage and cycle kinematic best practice at the elite international level, as well as the extent to which cycle characteristics are influenced by snow conditions, course profiles, and the type of events (e.g., sprints or marathons) is now required.

## Supporting information

S1 Data 10 km Classic macro-kinematic data. (XLSX)
